# Supplementary material for: Mechanisms governing the pioneering and redistribution capabilities of the non-classical pioneer PU.1
Source: Nat Commun. 2020 Jan 21;11:402. doi: 10.1038/s41467-019-13960-2 (PMC6972792; doi:10.1038/s41467-019-13960-2)
Supplement: Supplementary file 7 — Source data [file 41467_2019_13960_MOESM7_ESM.zip › Source_Data/Figure6/Figure6B_MotifScanOutput/FLI1_mutPU.1/homerResults/motif3.similar.html]

motif3

## Information for motif3

G
A
C
T
C
G
A
T
C
A
T
G
G
A
C
T
T
A
C
G
A
C
T
G
A
G
C
T
A
G
C
T
C
G
A
T
  
Reverse Opposite:  

G
C
T
A
T
C
G
A
T
C
G
A
A
G
T
C
A
T
G
C
C
T
G
A
G
T
A
C
G
C
T
A
C
T
G
A
  

|  |  |
| --- | --- |
| p-value: | 1e-899 |
| log p-value: | -2.072e+03 |
| Information Content per bp: | 1.721 |
| Number of Target Sequences with motif | 4554.0 |
| Percentage of Target Sequences with motif | 44.39% |
| Number of Background Sequences with motif | 5233.7 |
| Percentage of Background Sequences with motif | 13.73% |
| Average Position of motif in Targets | 102.1 +/- 48.9bp |
| Average Position of motif in Background | 98.5 +/- 62.2bp |
| Strand Bias (log2 ratio + to - strand density) | 0.1 |
| Multiplicity (# of sites on avg that occur together) | 1.29 |
| Motif File: | file (matrix) reverse opposite |

### Similar de novo motifs found

|  |  |  |  |  |  |  |  |
| --- | --- | --- | --- | --- | --- | --- | --- |
| Rank | Match Score | Redundant Motif | P-value | log P-value | % of Targets | % of Background | Motif file |
| 1 | 0.988 | T C G A G C T A T C G A T C G A A G T C A G T C C G T A A T G C G C T A C T A G T G A C T G C A | 1e-849 | -1957.138302 | 41.28% | 12.35% | motif file (matrix) |
| 2 | 0.983 | G C A T C T G A T G C A A G T C A G T C C T G A A G T C C G T A | 1e-794 | -1828.744558 | 46.36% | 16.57% | motif file (matrix) |
| 3 | 0.958 | G T C A T C G A T C G A A G T C A G T C C G T A A G T C C G T A C T A G T G A C G T C A G T C A G T C A T C G A | 1e-730 | -1682.563029 | 35.35% | 10.15% | motif file (matrix) |
| 4 | 0.917 | A G C T A G T C G C A T T C A G G A C T T C A G C T A G A G C T G C A T | 1e-668 | -1539.298342 | 48.72% | 20.40% | motif file (matrix) |
| 5 | 0.940 | A C T G C G A T C A G T C A T G A C T G G A T C C G A T A C T G A C G T A C T G A C T G A C G T A G C T | 1e-652 | -1503.109688 | 33.35% | 9.93% | motif file (matrix) |
| 6 | 0.972 | A C G T A C T G A G C T A C T G A C T G A C G T G A C T | 1e-634 | -1461.540797 | 31.29% | 8.92% | motif file (matrix) |
| 7 | 0.891 | G A T C A C G T A C T G A G C T A T C G T A C G A C G T | 1e-627 | -1444.523664 | 33.51% | 10.37% | motif file (matrix) |
| 8 | 0.892 | G A T C C G T A T C G A A G T C A G T C C G T A A T G C C G T A C T G A T G C A | 1e-595 | -1372.151181 | 51.33% | 23.88% | motif file (matrix) |
| 9 | 0.800 | G C A T C G T A C T G A A G T C T G A C C T A G G A T C | 1e-499 | -1149.043098 | 50.08% | 24.90% | motif file (matrix) |
| 10 | 0.895 | G T C A C T G A A G T C G A T C C T A G A G T C G C T A C T A G | 1e-497 | -1146.364538 | 46.46% | 21.95% | motif file (matrix) |
| 11 | 0.821 | G T C A G T C A T C G A A G T C G T A C C T A G G A T C G C T A T C A G G T C A | 1e-406 | -935.835066 | 36.01% | 15.78% | motif file (matrix) |
| 12 | 0.832 | G T C A T C A G T C G A T C G A C G T A C G T A C T G A A G T C G T A C G C T A A G T C G C T A T C A G | 1e-354 | -816.193571 | 21.00% | 6.61% | motif file (matrix) |
| 13 | 0.816 | G T C A T C G A T G C A T G A C G A T C G T C A G T A C G T C A A T G C G C A T A C G T A G T C G A T C | 1e-118 | -272.060122 | 5.85% | 1.47% | motif file (matrix) |
| 14 | 0.798 | G T A C C G A T T A C G T A G C A G T C A G T C T G C A A T G C C G T A | 1e-117 | -269.837566 | 15.18% | 7.39% | motif file (matrix) |
| 15 | 0.797 | C A G T C A G T A T C G C G A T C T A G C A T G A C G T G A C T C T G A T C A G G T C A C A G T C A G T | 1e-110 | -254.137030 | 6.73% | 2.04% | motif file (matrix) |
| 16 | 0.808 | C T A G A C G T A T C G A C T G A C G T A C G T T C G A A G C T C A T G G A T C | 1e-72 | -166.059288 | 3.76% | 0.99% | motif file (matrix) |
